# The frequency and severity of capecitabine-induced hypertriglyceridaemia in routine clinical practice: a prospective study

**DOI:** 10.1038/sj.bjc.6605807

**Published:** 2010-07-27

**Authors:** C O Michie, M Sakala, I Rivans, M W J Strachan, S Clive

**Affiliations:** 1 Edinburgh Cancer Centre, Western General Hospital, Crewe Road South, Edinburgh EH4 2XU, UK; 2 Metabolic Unit, Western General Hospital, Crewe Road South, Edinburgh EH4 2XU, UK

**Keywords:** capecitabine, triglycerides, hypertriglyceridaemia, lipids, dyslipidaemia

## Abstract

**Background:**

Capecitabine is known to rarely cause raised serum triglycerides (TG). In our centre, several patients receiving capecitabine developed raised TG levels corresponding to the ‘very high risk’ category for potentially serious acute pancreatitis.

**Methods:**

A fasting blood lipid screening protocol was introduced into clinical practice for patients receiving capecitabine. Patients with TGs >5 mmol l^−1^ were treated and followed up. An 18-month prospective audit was performed to establish the incidence and severity of capecitabine-induced hypertriglyceridaemia (CIHT).

**Results:**

A total of 304 patients received capecitabine for colorectal cancer between January 2008 and June 2009. Of these, 212 patients (70%) were screened and 8 (3.7%) developed clinically significant hypertriglyceridaemia requiring lipid-lowering therapy. Two of the eight patients had diabetes and one had pre-existing dyslipidaemia. One suffered cerebral infarction during chemotherapy. There were no cases of acute pancreatitis. Follow-up showed that serum TGs safely and rapidly returned to normal with appropriate treatment without discontinuation of capecitabine.

**Conclusions:**

This is the first prospective study evaluating CIHT. These results suggest that it should be classed as a ‘common’ undesired effect of capecitabine. Despite this, the incidence does not justify routine screening in all patients. Targeted screening in those with diabetes or pre-existing hyperlipidaemia is recommended, together with adoption of a clear management policy.

A number of anti-cancer drugs, such as tamoxifen and rapamycin, are known to cause abnormal lipid metabolism. Capecitabine is a prodrug of the cytotoxic agent 5-fluorouracil (5-FU), and is commonly used in the management of metastatic gastrointestinal (GI) and breast malignancies and as adjuvant therapy for colorectal carcinoma. It is associated with several adverse effects including diarrhoea, plantar-palmar erythrodysaesthesia and nausea.

Hypertriglyceridaemia has been associated with capecitabine treatment. In the manufacturer's product literature, National Cancer Institute Common Toxicity Criteria (NCI CTC) grade 3 (>5- to 10-fold increase) or 4 (>10-fold increase above upper limit of normal range (ULN)) hypertriglyceridaemia is listed as an uncommon (⩾0.1 to <1%) toxicity (Roche Pharmaceuticals, 2009). However, in five of the first main published capecitabine phase I studies in GI cancers (Budman *et al*, 1998; Cassidy *et al*, 1998; Mackean *et al*, 1998; Villalona-Calero *et al*, 1999; Pronk *et al*, 2000) involving a total of 143 patients, significant hypertriglyceridaemia was never reported. As it is thought to be an infrequent side effect and not currently tested for in routine practice, it is not reported in large phase III studies either (Cassidy *et al*, 2008).

Despite being listed as an uncommon toxicity, nine instances of grade 3/4 non-fasting hypertriglyceridaemia, at levels corresponding to the ‘high risk’ (>10 mmol l^−1^) or ‘very high risk’ (>20 mmol l^−1^) categories for development of potentially life-threatening acute pancreatitis, were noted anecdotally during 2007. All patients had been receiving capecitabine (either as 1250 mg m^−2^ twice daily on days 1–14 of 21-day cycle as monotherapy or 1000 mg m^−2^ in combination regimens) for colorectal cancer at the Edinburgh Cancer Centre.

In each of these cases, the hospital laboratory had initiated lipid testing after observing that routine pre-chemotherapy blood samples were frankly lipaemic. The highest chemotherapy-related non-fasting triglyceride (TG) level noted was 56 mmol l^−1^, in a patient with established diabetes (27 times the ULN).

To date, there have been five publications involving seven detailed case reports of severe capecitabine-induced hypertriglyceridaemia (CIHT) (Koutras *et al*, 2006; Kurt *et al*, 2006; Bar-Sela and Haim, 2009; Garg *et al*, 2009; Orphanos *et al*, 2010), with up to 20-fold reported increases from pre-treatment levels, all requiring interruption of capecitabine therapy. There have also been case reports of capecitabine-induced pancreatitis (Jones and Valero, 2003; Yucel and van Warmerdam, 2010) and of the association between CIHT and diabetes (Garg *et al*, 2009). The authors of the latter report state that patients receiving capecitabine therapy should have regular monitoring of lipid and glucose levels. Both of these tests are most meaningful when performed after an overnight fast. Logistically, performing regular fasting blood screening on all patients receiving such a commonly used cancer drug would require significant additional resource and clinical co-ordination. However, other than one small study in which 5 out of 12 patients developed CIHT (Stathopoulos *et al*, 2007), published data does not indicate whether the incidence and severity of this side effect support routine lipid testing.

Therefore, before considering widespread incorporation of lipid monitoring into clinical practice, the true frequency and severity of this side effect in routine practice needs to be established.

The main concern of an acutely raised serum TG level is acute pancreatitis. However, many patients are treated with capecitabine over a 6-month period, on an adjuvant basis, and chronic high levels could also have an impact on their general health and atherosclerosis risk.

## Study objectives

The primary study objective was to prospectively audit the incidence of severe CIHT when incorporating lipid testing into routine clinical practice for a trial period. The secondary objectives were to identify any specific high-risk groups that might be suitable for targeted screening, if screening the whole population was not felt to be justified or practical.

## Patients and methods

A management protocol for the timing of blood sampling and the management of hypertriglyceridaemia was formulated in collaboration with a local metabolic medicine specialist (MS; see Table 1 for protocol). Ethical approval for the audit was obtained from the Local Research Ethics Committee. All staff involved in the care of lower GI cancer patients receiving capecitabine, including general practitioners (GPs), were informed about the new lipid testing protocol and briefed as to the purpose of the audit and their individual responsibilities.

Patients who were yet to commence capecitabine therapy after 1 January 2008 were advised, as part of their pre-chemotherapy discussion with medical or nursing staff, that fasting serum lipid profiles would be required as part of their pre-chemotherapy work-up.

Fasting serum lipid profiles were performed along with routine pre-chemotherapy blood tests, at three pre-specified time points: before commencement of capecitabine, midway through therapy at cycle 3 or 4 and before the final cycle. Those with any abnormal levels (>2.1 mmol l^−1^) were to be followed up for 3 months after the end of chemotherapy, or more often if required.

Any patient with a fasting serum TG result >5.0 mmol l^−1^ during the course of chemotherapy was referred to the first author (COM) to ensure continuity and follow-up of future results. Data relating to patient demographics, oncology history, past medical history, concomitant medications, alcohol intake, renal function, body surface area, baseline blood pressure and family history of diabetes mellitus and hyperlipidaemia were collected. The patients, their oncologists and GPs were informed of the result and the recommendation for lipid-lowering therapy.

It was felt that the development of hypertriglyceridaemia should not interrupt capecitabine anti-cancer therapy; rather, that it be appropriately treated with oral fenofibrate (267 mg once daily), with close follow-up. In published case reports, patients did have capecitabine therapy withheld or interrupted; however, following discussion with our metabolic team, we did not feel that this was warranted, particularly given the importance of the chemotherapy.

## Results

**Figure 1:**
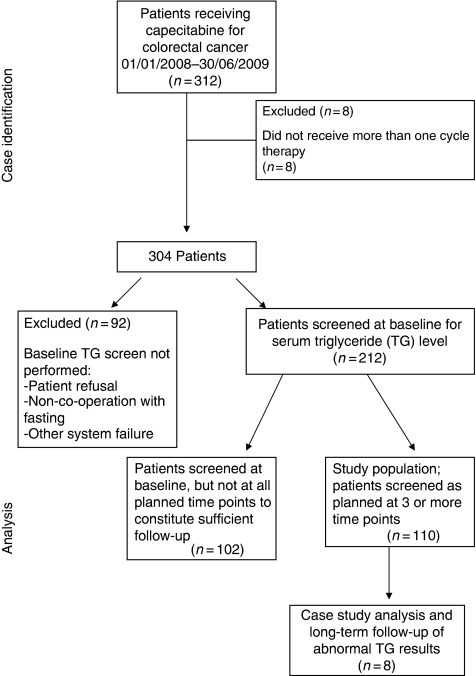
Patient flow diagram.

A total of 304 patients were treated with capecitabine (84 capecitabine alone, 220 in combination with oxaliplatin) for colorectal malignancies, with 1505 courses delivered during the 18-month observation period between 1 January 2008 and 30 June 2009. Figure 1 shows the flow of patients by means of a Consort diagram, and the patient characteristics of those who developed CIHT are summarised in Table 2.

A total of 212 patients (70%) were screened at baseline for hypertriglyceridaemia as part of this study. Of these, 30 were known to have pre-existing diabetes (14.1%). Unfortunately, only 36% of patients were screened at all three time points during therapy as planned, because of patient refusal or non-compliance with fasting, appointment scheduling difficulties making fasting problematic or other failures in the system.

Six patients were found to have raised TGs before commencing capecitabine and were referred directly to their GP to start corrective treatment. None of those who had TG levels repeated during chemotherapy (*n*=3) had any significant rise in their TGs with concomitant fibrate administration.

All eight patients who developed CIHT during the study period were being treated with the combination regimen of capecitabine and oxaliplatin. This was in contrast to the group of nine patients noted to have developed CIHT during 2007, which led to the development of the study. Of these, 66% had been receiving capecitabine monotherapy. However, by the time of commencement of the study in 1 January 2008, the combination regimen had been widely adopted in our unit to be the standard of care for colorectal cancer, and indeed only 26% of the entire screened population in the prospective study had received capecitabine alone.

In the group of eight patients with CIHT, only one had previously been diagnosed with dyslipidaemia and was receiving statin therapy. Two patients had previously had lipid screening, which had been normal. No patients had a history of ischaemic heart disease (given the side-effect profile of capecitabine) and one had a history of treated hypertension. All patients had a normal creatinine clearance and none were grossly overweight, but two consumed more than the safe recommended limit of alcohol. The median age was 54 years. Four had had previous separate lines of capecitabine chemotherapy for their malignancy.

Six (3%) of the non-diabetic patients developed significantly raised TGs and were commenced on fenofibrate as per protocol. Two patients with non-insulin-dependent diabetes (6%) developed hypertriglyceridaemia and there was also a definite trend towards moderately raised TGs in the diabetic group; 14 (47%) were shown to have TG levels above the upper limit of the normal range (>2.1 mmol l^−1^). The median fasting TG level for these diabetic patients with moderately raised TG levels (between 2.1 and 5 mmol l^−1^) at any time point was 3.29 mmol l^−1^.

The patients with the highest TG levels were those with mild or moderate derangement of their TG levels at baseline, before commencement of capecitabine therapy. There was only one case of NCI CTC grade 4 TGs (defined as >21 mmol l^−1^, >10 times the ULN) in this audit period. This is in contrast to the multiple grossly deranged levels observed anecdotally in the preceding year. This may be a reflection of the prospective pre- and early-treatment testing, and the implementation of a standard management policy for raised TGs.

**Figure 2:**
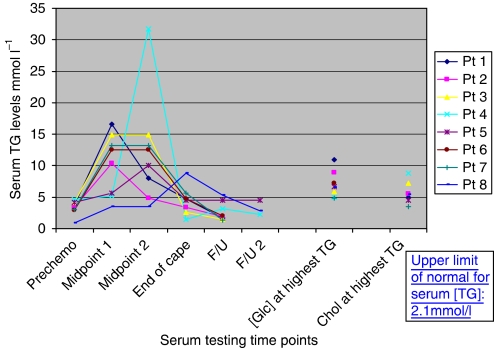
Triglyceride (TG) levels over time in eight patients with capecitabine-induced hypertriglyceridaemia requiring lipid-lowering therapy. The colour reproduction of this figure is available on the html full text version of the manuscript.

The TG levels of the eight patients with severely raised TG are displayed graphically in Figure 2. Levels of serum glucose and total cholesterol at the time of the highest serum TG level are also shown for each patient. There was no evidence of hepatic steatosis in any patients with CIHT.

### Outcomes

Treatment with standard fenofibrate (267 mg daily) was successful with complete TG level normalisation in all but one case. Normalisation was also sustained at 3 months of follow-up. In one patient, the serum TG level doubled on discontinuation of fenofibrate therapy and had to be restarted. Three patients remained on fenofibrate therapy at their last clinic visit, but five had discontinued therapy after 3 months with a normal serum TG level, as per protocol. Some patients had frequent follow-up serum TG sampling for up to 1 year, which was at their clinicians discretion, and these remained normal.

One patient diagnosed with CIHT had a cerebral infarction after cycle 2 of capecitabine/oxaliplatin. His TGs had risen significantly since starting of treatment (16.6 mmol l^−1^ fasting from pre-treatment non-fasting level of 10.25 mmol l^−1^). There were no known cases of acute pancreatitis in the patients audited.

## Discussion

This is the first study to our knowledge that prospectively evaluated changes in fasting serum TGs with capecitabine therapy in standard oncology practice. The frequency of CIHT observed in our population who were screened at any time was 3.7%, which would be classed as a ‘common’ undesirable effect (>1% and <10%) rather than ‘uncommon’, as currently stated in the Summary of Product Characteristics (Roche Pharmaceuticals, 2009). It is possible that this percentage would have been higher had there not been a robust policy for early management of hypertriglyceridaemia, including those detected at baseline. There was a trend towards increased risk of CIHT in patients with diabetes, but no other clear risk factors were identified, other than those with mild-to-moderate TG derangement at baseline.

In 80% of the cases requiring lipid-lowering therapy, CIHT responded well to fibrate treatment. There were no acute serious clinical consequences of CIHT in our population, although our early-treatment policy may have been a factor. In the case of the patient with the ischaemic stroke while on capecitabine/oxaliplatin chemotherapy, his pre-existing non-fasting hypertriglyceridaemia may have increased the risk, but the additional impact of the further rise in TG on capecitabine is unknown.

This was the first time that fasting lipid screening tests had been incorporated into standard chemotherapy practice in our cancer centre. All staff groups were informed about the new lipid screening policy, and information routinely given to each new patient starting treatment. Despite this, lipid testing was only performed in 70% of patients and at all three time points in 36%. This highlights the difficulties in performing non-standard tests outside a formal clinical trial setting. A particular difficulty in preventing patient dropout was the requirement to fast for 12 hours; this can be disruptive to patients’ lifestyles and complicate appointment scheduling, particularly for patients with diabetes. Undoubtedly, however, there were practical benefits in having a clear protocol to follow when raised TGs were identified, as this is something oncologists do not normally treat.

We acknowledge that this study would undoubtedly be more succinct if all patients were simply receiving capecitabine monotherapy, rather than the majority also receiving oxaliplatin. However, this reflected the change in our practice at the time to use combination therapy, as standard, and it would clearly be unethical to withhold oxaliplatin in the interests of such a project. In addition, it would take a considerable amount of time to obtain sufficient numbers of patients receiving capecitabine alone to obtain a meaningful conclusion. Importantly, hypertriglyceridaemia, to our knowledge, has not previously been reported with oxaliplatin alone and specifically not with the FOLFOX (combination oxaliplatin with infusional 5-FU) regimen, which has been commonly used worldwide since ∼2004. Nevertheless, we cannot exclude an interaction between the two drugs.

The underlying mechanism leading to CIHT is currently unknown. Hypertriglyceridaemia has not been reported as a side effect of 5-FU, suggesting that the effect is related to the capecitabine itself or its metabolites before the formation of 5-FU. Given its perceived rarity, the underlying mechanism has not so far been studied in detail (Bar-Sela and Haim, 2009), but one theory postulated is that capecitabine may reduce the activity of lipoprotein lipase and hepatic TG lipase (Stathopoulos *et al*, 2007; Garg *et al*, 2009).

As a result of our audit, we concluded that continuing routine lipid screening of all our patients receiving capecitabine-containing chemotherapy regimens is not justified.

However, it is important that oncology teams are aware of this potentially medically important side effect, and consideration should be given to lipid monitoring in patients with established diabetes or dyslipidaemia (Stathopoulos *et al*, 2007; Bar-Sela and Haim, 2009; Garg *et al*, 2009). We would suggest serum lipid sampling for those patients at baseline and after 2 or 3 cycles of capecitabine, and recommend having an agreed policy for the management of hypertriglyceridaemia when it is detected. Importantly, and in contrast to previous published case reports, our protocol did not involve discontinuation or interruption of capecitabine chemotherapy due to CIHT and all patients in our group were safely treated with immediate lipid-lowering therapy (with the sole exception of the patient with cerebral infarction, whose capecitabine was discontinued on that basis) and close follow-up of their serum TG levels.

## Figures and Tables

**Table 1:** Management protocol for raised serum triglycerides

**Abnormal level**	**Action**
Serum TG level >5 mmol l^−1^ pre chemotherapy	• Refer directly to GP for investigation and/or management
Fasting serum TG level 5–10 mmol l^−1^ during chemotherapy	• Calculate CVD risk prediction using validated prediction charts (from Williams *et al*, 2004); if CVD risk >20% over next 10 years, start fenofibrate as given below, if cancer-related prognosis is sufficient to justify this (>3 months)
Serum TG level >10 mmol l^−1^ during chemotherapy	• Commence oral fenofibrate (267 mg) daily (unless any contraindications) • Inform patient and GP of result and advice • Repeat serum TG level after 1–2 months to ensure normalisation of TG level • Discontinue fenofibrate 3 months after end of chemotherapy if TG improves to <2.1 mmol l^−1^ and CVD risk score is <20% • Advise further repeat lipid profiling 3 months after discontinuation of fenofibrate
Grossly elevated TG level (>15 mmol l^−1^) at any time	• As per serum TG level >10 mmol l^−1^, but also refer directly to metabolic team for investigation

Abbreviations: CVD=cardiovascular disease; GP=general practitioner; TG=triglycerides.

**Table 2:** Characteristics of patients with CIHT

**Variable**	**Patients with CIHT requiring treatment (*n*=8)**
Median age (years)	54 (range 44–69)
Site of primary tumour	Colon: 5; rectum: 2; unknown: 1
Chemotherapy regimen	Capecitabine/oxaliplatin combination: 8 patients Capecitabine alone: 0 (total screened population receiving capecitabine alone: 56 (26%))
Treatment intent	Adjuvant: 5 Palliative: 3
History of diabetes	2 Patients, both non-insulin-dependent (total number screened diabetic patients=30)
Known history of dyslipidaemia or any lipid-lowering therapy	Dyslipidaemia: 1 patient receiving atorvastatin therapy before chemotherapy, but no previous fibrate therapy Lipids never previously tested: 5 Lipids previously tested but normal: 2
Previous history of ischaemic heart disease	None
Previous history of hypertension	1 Patient on anti-hypertensive therapy
Previous line of capecitabine therapy	4 Patients
Renal function	All patients with documented creatinine clearance of >60 ml min^−1^
Alcohol consumption	Never consume: 3 Moderate consumption: 3 Above safe recommended limit: 2
Median body surface area (m^2^)	1.84 (range 1.64–2.28)

Abbreviation: CIHT=capecitabine-induced hypertriglyceridaemia.
